# 2-({4-[(1*H*-Imidazol-2-ylsulfanyl)methyl]-2,5-dimethylbenzyl}sulfanyl)-1*H*-imidazole

**DOI:** 10.1107/S1600536810039607

**Published:** 2010-10-09

**Authors:** Malcolm J. Applewhite, Storm V. Potts

**Affiliations:** a Department of Chemistry and Polymer Science, Stellenbosch University, Private Bag X1, Matieland, 7602, South Africa

## Abstract

The title compound, C_16_H_18_N_4_S_2_, was prepared by the substitution reaction of two equivalents of 2-mercaptoimidazole for every bromine substituent of 1,4-bis­(bromo­meth­yl)-2,5-dimethyl­benzene. The mol­ecule is located on a crystallographic centre of inversion and therefore adopts a *trans* configuration with regards to the orientation of the two sulfur atoms. An inter­molecular N—H⋯N hydrogen bond forms layers of mol­ecules parallel to (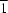
03). The dihedral angle between the central and terminal rings is 174.8 (2)°.

## Related literature

For related structures, see: Fan *et al.* (2003 ▶); Voo *et al.* (2003 ▶).
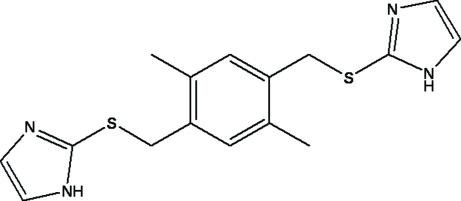


## Experimental

### 

#### Crystal data

C_16_H_18_N_4_S_2_
*M* *_r_* = 330.46
Monoclinic, 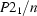

*a* = 6.169 (3) Å
*b* = 9.491 (5) Å
*c* = 13.722 (8) Å
β = 95.392 (8)°
*V* = 799.8 (8) Å^3^
*Z* = 2
Mo *K*α radiation
μ = 0.33 mm^−1^
*T* = 100 K
0.2 × 0.14 × 0.07 mm

#### Data collection

Bruker APEX CCD area-detector diffractometer
Absorption correction: multi-scan (*SADABS*; Bruker, 2009 ▶) *T*_min_ = 0.945, *T*_max_ = 0.977
4896 measured reflections
1875 independent reflections
1154 reflections with *I* > 2sσ(*I*)
*R*_int_ = 0.075

#### Refinement

*R*[*F* ^2^ > 2σ(*F* ^2^)] = 0.058
*wR*(*F* ^2^) = 0.149
*S* = 1.01
1875 reflections
101 parameters
H-atom parameters constrained
Δρ_max_ = 0.67 e Å^−3^
Δρ_min_ = −0.39 e Å^−3^

### 

Data collection: *APEX2* (Bruker, 2009 ▶); cell refinement: *SAINT* (Bruker, 2009 ▶); data reduction: *SAINT*; program(s) used to solve structure: *SHELXS97* (Sheldrick, 2008 ▶); program(s) used to refine structure: *SHELXL97* (Sheldrick, 2008 ▶); molecular graphics: *X-SEED* (Barbour, 2001 ▶); software used to prepare material for publication: *X-SEED*.

## Supplementary Material

Crystal structure: contains datablocks I, New_Global_Publ_Block. DOI: 10.1107/S1600536810039607/bt5352sup1.cif

Structure factors: contains datablocks I. DOI: 10.1107/S1600536810039607/bt5352Isup2.hkl

Additional supplementary materials:  crystallographic information; 3D view; checkCIF report

## Figures and Tables

**Table 1:** Hydrogen-bond geometry (Å, °)

*D*—H⋯*A*	*D*—H	H⋯*A*	*D*⋯*A*	*D*—H⋯*A*
N1—H1⋯N4^i^	0.88	1.93	2.791 (4)	165

Symmetry code: (i) 
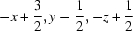
.

**Fig. 1.**
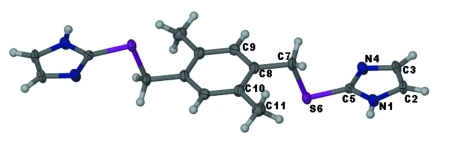
The molecular structure of the title compound which has 50% probability ellipsoids. The trans relationship between the sulfur atoms can clearly be seen.

**Fig. 2.**
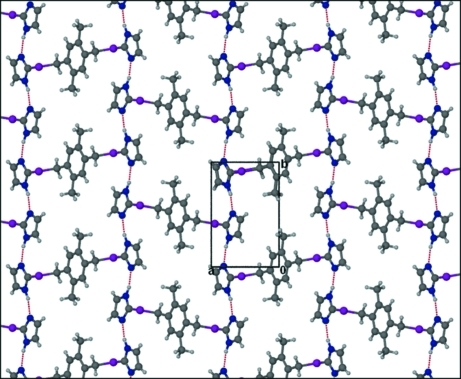
The crystal packing viewed down the c axis shows N—H···N hydrogen bonding occurring to form chains throughout a layer of the crystal structure.

## supplementary crystallographic information

### Comment

The molecule adopts a *trans* configuration with regards to the orientation of the two sulfur atoms.

### Experimental

2-Mercaptoimidazole (1.60 g, 16 mmol) was added to 1,4-bis(bromomethyl)-2,5-dimethylbenzene (1.30 g, 4 mmol) in 200 mL of MeOH. The resulting solution was refluxed for 24 h.

The solvent was then removed *in vacuo* and K_2_CO_3_ (6.91 g, 50 mmol) in 100 mL of H_2_O was added. The solution was stirred until the product precipitated. The white solid was then filtered, washed with 100 mL of H_2_O and left to air dry.

### Refinement

All hydrogen atoms  were refined in calculated positions, using a riding model (C–H*_ar_* = 0.95 Å; C—H = 0.99 Å; N–H = 0.88 Å) with U(H) set to 1.2U_eq_ of the parent atom (1.5 for methyl H atoms).

### Crystal data

**Table:**

C_16_H_18_N_4_S_2_	*F*(000) = 348
*M**_r_* = 330.46	*D*_x_ = 1.372 Mg m^−^^3^
Monoclinic, *P*2_1_/*n*	Mo *K*α radiation, λ = 0.71073 Å
Hall symbol: -P2yn	Cell parameters from 394 reflections
*a* = 6.169 (3) Å	θ = 2.6–28.2°
*b* = 9.491 (5) Å	µ = 0.33 mm^−^^1^
*c* = 13.722 (8) Å	*T* = 100 K
β = 95.392 (8)°	Shard, colourless
*V* = 799.8 (8) Å^3^	0.2 × 0.14 × 0.07 mm
*Z* = 2	

### Data collection

**Table:**

Bruker APEX CCD area-detector diffractometer	1875 independent reflections
Radiation source: fine-focus sealed tube	1154 reflections with *I* > 2sσ(*I*)
graphite	*R*_int_ = 0.075
ω scans	θ_max_ = 28.2°, θ_min_ = 2.6°
Absorption correction: multi-scan (*SADABS*; Bruker, 2009)	*h* = −8→7
*T*_min_ = 0.945, *T*_max_ = 0.977	*k* = −12→6
4896 measured reflections	*l* = −17→18

### Refinement

**Table:**

Refinement on *F*^2^	Primary atom site location: structure-invariant direct methods
Least-squares matrix: full	Secondary atom site location: difference Fourier map
*R*[*F*^2^ > 2σ(*F*^2^)] = 0.058	Hydrogen site location: inferred from neighbouring sites
*wR*(*F*^2^) = 0.149	H-atom parameters constrained
*S* = 1.01	*w* = 1/[σ^2^(*F*_o_^2^) + (0.0617*P*)^2^] where *P* = (*F*_o_^2^ + 2*F*_c_^2^)/3
1875 reflections	(Δ/σ)_max_ < 0.001
101 parameters	Δρ_max_ = 0.67 e Å^−^^3^
0 restraints	Δρ_min_ = −0.39 e Å^−^^3^

### Special details

**Table:**

Geometry. All e.s.d.'s (except the e.s.d. in the dihedral angle between two l.s. planes) are estimated using the full covariance matrix. The cell e.s.d.'s are taken into account individually in the estimation of e.s.d.'s in distances, angles and torsion angles; correlations between e.s.d.'s in cell parameters are only used when they are defined by crystal symmetry. An approximate (isotropic) treatment of cell e.s.d.'s is used for estimating e.s.d.'s involving l.s. planes.
Refinement. Refinement of *F*^2^ against ALL reflections. The weighted *R*-factor *wR* and goodness of fit *S* are based on *F*^2^, conventional *R*-factors *R* are based on *F*, with *F* set to zero for negative *F*^2^. The threshold expression of *F*^2^ > σ(*F*^2^) is used only for calculating *R*-factors(gt) *etc*. and is not relevant to the choice of reflections for refinement. *R*-factors based on *F*^2^ are statistically about twice as large as those based on *F*, and *R*- factors based on ALL data will be even larger.

### Fractional atomic coordinates and isotropic or equivalent isotropic displacement parameters (Å^2^)

**Table:**

	*x*	*y*	*z*	*U*_iso_*/*U*_eq_	
N1	0.7634 (4)	0.7794 (3)	0.28821 (19)	0.0230 (7)	
H1	0.7160	0.6949	0.2705	0.028*	
C2	0.8959 (5)	0.8119 (4)	0.3693 (2)	0.0249 (8)	
H2	0.9548	0.7482	0.4182	0.030*	
C3	0.9285 (5)	0.9529 (4)	0.3674 (2)	0.0257 (8)	
H3	1.0158	1.0050	0.4154	0.031*	
N4	0.8168 (4)	1.0086 (3)	0.28587 (18)	0.0250 (7)	
C5	0.7181 (5)	0.8997 (4)	0.2400 (2)	0.0205 (7)	
S6	0.55288 (13)	0.91254 (10)	0.12971 (6)	0.0241 (3)	
C7	0.2861 (5)	0.9423 (4)	0.1750 (2)	0.0275 (8)	
H7A	0.2403	0.8586	0.2109	0.033*	
H7B	0.2914	1.0246	0.2195	0.033*	
C8	0.1309 (5)	0.9690 (4)	0.0855 (2)	0.0233 (8)	
C9	0.0933 (5)	1.1077 (4)	0.0551 (2)	0.0243 (8)	
H9	0.1587	1.1819	0.0939	0.029*	
C10	0.0364 (5)	0.8586 (4)	0.0300 (2)	0.0242 (8)	
C11	0.0624 (6)	0.7085 (4)	0.0609 (3)	0.0320 (9)	
H11A	−0.0269	0.6900	0.1149	0.048*	
H11B	0.2156	0.6898	0.0825	0.048*	
H11C	0.0154	0.6469	0.0055	0.048*	

### Atomic displacement parameters (Å^2^)

**Table:**

	*U*^11^	*U*^22^	*U*^33^	*U*^12^	*U*^13^	*U*^23^
N1	0.0274 (15)	0.0207 (16)	0.0199 (14)	−0.0032 (13)	−0.0031 (11)	−0.0010 (12)
C2	0.0244 (19)	0.030 (2)	0.0189 (17)	0.0024 (15)	−0.0067 (14)	0.0045 (15)
C3	0.0272 (19)	0.032 (2)	0.0163 (16)	−0.0024 (16)	−0.0055 (13)	0.0015 (15)
N4	0.0290 (16)	0.0248 (16)	0.0201 (14)	−0.0021 (13)	−0.0035 (12)	−0.0009 (13)
C5	0.0209 (17)	0.0212 (18)	0.0190 (16)	0.0042 (15)	−0.0002 (13)	−0.0013 (14)
S6	0.0221 (5)	0.0314 (5)	0.0177 (4)	0.0031 (4)	−0.0038 (3)	−0.0002 (4)
C7	0.0235 (18)	0.039 (2)	0.0194 (16)	0.0085 (16)	−0.0035 (13)	0.0023 (16)
C8	0.0189 (17)	0.032 (2)	0.0185 (16)	0.0028 (15)	0.0004 (13)	0.0008 (15)
C9	0.0182 (17)	0.029 (2)	0.0249 (17)	0.0009 (15)	0.0002 (13)	−0.0051 (15)
C10	0.0229 (18)	0.0268 (19)	0.0230 (17)	0.0031 (15)	0.0017 (14)	0.0031 (15)
C11	0.034 (2)	0.027 (2)	0.036 (2)	0.0035 (17)	0.0045 (16)	0.0035 (17)

### Geometric parameters (Å, °)

**Table:**

N1—C5	1.336 (4)	C7—H7A	0.9900
N1—C2	1.354 (4)	C7—H7B	0.9900
N1—H1	0.8800	C8—C10	1.391 (5)
C2—C3	1.353 (5)	C8—C9	1.394 (5)
C2—H2	0.9500	C9—C10^i^	1.389 (4)
C3—N4	1.364 (4)	C9—H9	0.9500
C3—H3	0.9500	C10—C9^i^	1.389 (4)
N4—C5	1.327 (4)	C10—C11	1.491 (5)
C5—S6	1.748 (3)	C11—H11A	0.9800
S6—C7	1.835 (3)	C11—H11B	0.9800
C7—C8	1.506 (4)	C11—H11C	0.9800
			
C5—N1—C2	107.1 (3)	S6—C7—H7B	110.6
C5—N1—H1	126.5	H7A—C7—H7B	108.7
C2—N1—H1	126.5	C10—C8—C9	119.8 (3)
C3—C2—N1	106.8 (3)	C10—C8—C7	121.4 (3)
C3—C2—H2	126.6	C9—C8—C7	118.6 (3)
N1—C2—H2	126.6	C10^i^—C9—C8	122.4 (3)
C2—C3—N4	109.6 (3)	C10^i^—C9—H9	118.8
C2—C3—H3	125.2	C8—C9—H9	118.8
N4—C3—H3	125.2	C9^i^—C10—C8	117.8 (3)
C5—N4—C3	105.1 (3)	C9^i^—C10—C11	119.8 (3)
N4—C5—N1	111.5 (3)	C8—C10—C11	122.4 (3)
N4—C5—S6	124.1 (3)	C10—C11—H11A	109.5
N1—C5—S6	124.5 (3)	C10—C11—H11B	109.5
C5—S6—C7	100.70 (15)	H11A—C11—H11B	109.5
C8—C7—S6	105.7 (2)	C10—C11—H11C	109.5
C8—C7—H7A	110.6	H11A—C11—H11C	109.5
S6—C7—H7A	110.6	H11B—C11—H11C	109.5
C8—C7—H7B	110.6		
			
C5—N1—C2—C3	−0.4 (4)	C5—S6—C7—C8	174.8 (2)
N1—C2—C3—N4	0.2 (4)	S6—C7—C8—C10	83.0 (3)
C2—C3—N4—C5	0.1 (4)	S6—C7—C8—C9	−93.0 (3)
C3—N4—C5—N1	−0.4 (4)	C10—C8—C9—C10^i^	−0.2 (5)
C3—N4—C5—S6	−179.6 (2)	C7—C8—C9—C10^i^	175.9 (3)
C2—N1—C5—N4	0.5 (4)	C9—C8—C10—C9^i^	0.2 (5)
C2—N1—C5—S6	179.7 (2)	C7—C8—C10—C9^i^	−175.8 (3)
N4—C5—S6—C7	−90.1 (3)	C9—C8—C10—C11	−177.2 (3)
N1—C5—S6—C7	90.8 (3)	C7—C8—C10—C11	6.9 (5)

Symmetry codes: (i) −*x*, −*y*+2, −*z*.

### Hydrogen-bond geometry (Å, °)

**Table:**

*D*—H···*A*	*D*—H	H···*A*	*D*···*A*	*D*—H···*A*
N1—H1···N4^ii^	0.88	1.93	2.791 (4)	165

Symmetry codes: (ii) −*x*+3/2, *y*−1/2, −*z*+1/2.
